# Risk Assessment of Heavy Metals Pollution in Agricultural Soils of Siling Reservoir Watershed in Zhejiang Province, China

**DOI:** 10.1155/2013/590306

**Published:** 2013-09-15

**Authors:** Naveedullah, Muhammad Zaffar Hashmi, Chunna Yu, Hui Shen, Dechao Duan, Chaofeng Shen, Liping Lou, Yingxu Chen

**Affiliations:** ^1^Department of Environmental Engineering, College of Environmental and Resource Sciences, Zhejiang University, Hangzhou 310058, China; ^2^Center for Biomedicine and Health, Hangzhou Normal University, Hangzhou 311121, China

## Abstract

Presence of heavy metals in agriculture soils above the permissible limit poses threats to public health. In this study, concentrations of seven metals were determined in agricultural soils from Yuhang county, Zhejiang, China. Multivariate statistical approaches were used to study the variation of metals in soils during summer and winter seasons. Contamination of soils was evaluated on the basis of enrichment factor (EF), geoaccumulation index (*I*_geo_), contamination factor (*C*_*f*_), and degree of contamination (*C*_deg_). Heavy metal concentrations were observed higher in winter as compared to summer season. Cr and Cd revealed random distribution with diverse correlations in both seasons. Principal component analysis and cluster analysis showed significant anthropogenic intrusions of Zn, Cd, Pb, Cr, and Cu in the soils. Enrichment factor revealed significant enrichment (EF > 5) of Zn, Cd, and Pb, whereas geoaccumulation index and contamination factor exhibited moderate to high contamination for Zn, Cr, Cd, and Pb. In light of the studied parameters, permissible limit to very high degree of contamination (*C*_deg_ > 16) was observed in both seasons.

## 1. Introduction

Soil is an important compartment receiving a significant amount of pollutants from different sources every year. Generally, soil not only serves as sink for the chemical pollutants but also acts as a natural buffer by controlling the transport of chemical elements and substances to the environment [1]. Heavy metals are found ubiquitously in both polluted and unpolluted soil layers of many ecological systems. These heavy metals cannot be degraded or destroyed but only are accumulated in soil, water, and sediments. Heavy metals in soils may either be found naturally or generated from anthropogenic activities. Natural sources include atmospheric emissions from volcanoes, transport of continental dusts, and weathering of metal-enriched rocks [2]. However, anthropogenically origin sources related to the metal-enriched sewage sludges in agriculture, combustion, live stock manures, application of metal based pesticides, electronics (manufacture, use, and disposal), volcanic eruptions, forest fires, industrial processes, municipal wastes, and agricultural activities [3, 4].

The contamination of agricultural soil by toxic elements such as heavy metals attracts the interest of people not only because metals can build up in the soil but also because metals can be accumulated in crops, where they cause significant potential risk to human health [5, 6]. In developing countries, due to rapid industrialization, excessive application of metals and synthetic chemicals in the terrestrial environment coupled with deficient environmental management have led to a large-scale pollution in the environment. Soils contaminated by heavy metals from agricultural activities have raised serious concern in recent decades regarding potential risk to human health through the direct intake, bioaccumulation through food chain, and their impacts on ecological system [7, 8]. Essential heavy metals (copper (Cu), zinc (Zn), and manganese (Mn)) as well as nonessential heavy metals (cadmium (Cd), chromium (Cr), manganese (Mn), and lead (Pb)) are considered highly toxic for human and aquatic life [9]. Recent data revealed that adverse health effects of cadmium and lead exposure may occur at lower exposure levels than previously anticipated, primarily in the form of kidney damage, bone effects and fractures, and neurotoxic effects in children [10]. A specific amount of Cr (III) is needed for normal body functions, while its high concentrations along with Cr (IV) may cause toxicity, including liver and kidney problems and genotoxic carcinogen [11]. In the Earth crust, iron (Fe) is the most abundant metal and is essential to all organisms, except for few bacteria. Its excess in the body causes liver and kidney damage [12]. A seasonal succession of different elements can be a key factor in determining seasonal variation of metal concentrations in Siling reservoir and its relationships with environmental factors [13]. Keeping in view the important role of these heavy metals in environmental pollution and the possibility of their presence in Siling reservoir, we designed the present study. Our main objective was to peep into the possible role of seasonal variation in accumulation of these metals in the watershed areas of Siling reservoir.

Several studies have been conducted on the accumulation of metals in soils under different land uses in China [14–19]. However, past studies focused mainly on the metal contaminations of soils affected by farming, industry, or urban development. There have been less information regarding the metal contamination of soils in ecologically sensitive areas such as reservoir areas, which are the source sites of drinking water. Siling reservoir watershed is one of the typical small watersheds in Tiaoxi River watershed and is the main source of Taihu Lake, which is the second largest fresh water lake in China. The data provided in this study are considered important for reservoir management, since the Siling reservoir serves as one of the main drinking water sources for the people of Yuhang County, Hangzhou City. Agriculture is the dominant land-use and large amounts of agrochemicals have been applied to the farming areas of this region. Metals and pesticides in soils can reach the aquatic ecosystems by leaching, soil erosion, and surface runoff. Protection of the soil quality in the watershed of the Siling reservoir is of great importance for conserving the water quality in the reservoir.

The main objectives of the study were to observe any possible seasonal changes in the distribution of selected metals (Zn, Cu, Mn, Fe, Cr, Cd, and Pb) in soils in the watershed of the reservoir, any ecological and health related risk of selected metals to the inhabitants, and then to identify their natural and anthropogenic sources by multivariate statistical methods. The potential ecological risk index, which may be used as a diagnostic tool for determining the degree of pollution in the soil, was also assessed using the geoaccumulation index (*I*_geo_), enrichment factor (EF), contamination factor (*C*_*f*_), and degrees of contamination (*C*_deg⁡_). It is hoped that the study would provide a baseline data regarding the distribution and accumulation of the selected metals in the soil and would help reduce the contamination in the Siling watershed by identifying the major pollution sources. Furthermore, for the best management of the native soil resources, it is very important to have information about the pollution hazards and heavy metals concentration of the study area.

## 2. Materials and Methods

### 2.1. Site Description

**Figure 1:**
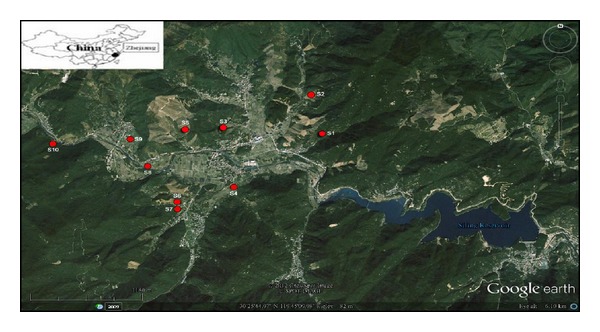
Location map of the sampling points in the study area.

Siling reservoir is located in the northeast of Hangzhou city, Zhejiang province, China (latitude 30.419~30.434, longitude 119.706~119.782) (Figure 1). Siling reservoir is the main drinking water source for Yuhang county, Hangzhou city. The catchments area of the reservoir is 80 km^2^ (approx.), while the length is 25 km (approx.), with maximum height of 600 m. The designed capacity of the reservoir is 2.8 × 10^7^ m^3^, with a depth of 78.03 m. Siling reservoir is surrounded by Jurassic mountains composed of rhyolite porphyry, tuff, and siltstone rocks. We determined soil texture in this area and the dominant soil class is loam. Land use types of the Siling watershed are agricultural, orchard, and forest land, which together accounts for about 90% of the total area. Tea, bamboo, pear and vegetables are major crops grown in the area. Soil of the study area is strongly acidic in nature showing mean pH values from 4.57 to 6.20 and organic matter content ranged from 4.03% to 5.50%. Nitrogen (urea), phosphate (superphosphate), and potash fertilizers, with organic manure, are applied to the farming areas of the reservoir. In the surrounding of the reservoir, there are many scenic spots, providing recreational facilities for the local public. Episodic runoff events from the agricultural and forest areas also drain into the reservoir. Algal bloom in the Siling reservoir has therefore extended coverage and persists throughout the summer season, which seriously affects the reservoir as a service of drinking water supply. 

### 2.2. Sample Collection and Preservation

Soil samples were collected in triplicates in the watershed of the reservoir during summer and winter 2012. Ten sampling points were selected from the potential agricultural fields in the watershed of the reservoir (Figure 1). The composite top surface soil samples (0–20 cm deep) were collected randomly in precleaned self-locking polythene bags, which were placed in airtight large plastic containers. Any foreign material/debris from the soil samples was manually removed during the sample collection. The soil samples were air-dried, grounded, homogenized, and sealed in clean polythene bags and refrigerated [20].

### 2.3. Sample Preparation and Analysis

Soil samples were processed to assess the total concentrations of the metals. To estimate the total metal contents, 1-2 g dried soil sample was digested in a microwave system using acid mixture (9 mL 6 M HNO_3_ + 3 mL 5 M HCl) [21]. For the determination of total heavy metals (e.g., Zn, Cu, Mn, Fe, Cr, Cd, and Fe), the extraction was carried out in Teflon containers provided with screw stoppers as mentioned by Tessier et al. [22]. For each digestion, a blank was also prepared with the same amount of acids without soil sample. The digested samples were then filtered through the fine filters and volume was raised up to 50 mL with deionized water and stored at 4°C. The acid-extract of the soil samples was analyzed using flame atomic absorption spectrophotometer (Shimadzu AA-670, Japan) under standard analytical conditions. Standard curve line method was used for the quantification of selected metals and the samples were diluted whenever required [20, 23]. Standard reference material was also used to ensure the reliability of the metal data (SRM 2711). Analytical grade chemicals were used throughout the study without any further purification. To prepare all the reagents and calibration standards, double distilled water was used. The metal standards were prepared from stock solution of 1000 mg L^−1^ by successive dilutions. The glasswares were washed with dilute nitric acid followed by several times washing with distilled water. All measurements were made in triplicates. Organic matter contents were determined by soil ignition at a temperature of 450°C [24]. Soil pH was measured in a soil deionized water suspension (soil : water, 1 : 2.5 by volume) by a calibrated pH meter [25]. Soil organic matter was determined according to the method of Bao [26].

### 2.4. Statistical Analyses

Statistical methods were applied to analyze the data in terms of its distribution and correlation among the studied parameters. Statistica version 5.5 software was used for statistical analyses of the metal data. Basic statistical parameters such as minimum, maximum, mean, standard deviation (SD), and standard error (SE) were computed along with correlation analysis, while multivariate statistics in terms of principal component analysis (PCA) and cluster analysis (CA) were also carried out [27, 28]. PCA was carried using varimax normalized rotation on the dataset and the CA was applied to the standardized matrix of samples, using Ward's method. CA was used to discover a system of organizing variables where each cluster shares properties in common and thus to make it cognitively easier to predict mutual properties based on an overall group membership.

To evaluate the magnitude of contaminants in the environment, the enrichment factors (EF) were computed by relating the abundance of species in source material to that found in the Earth's crust [29, 30]. EFs are usually taken as double ratios of the target metal and a reference metal in the examined soil and Earth crust. Usually, Al, Mg, Ca, Mn, and Fe are used as the reference. In our study, EFs were calculated using Mn as the reference, using the following equation:

(1) $$\begin{array}{c} \text{EF}=\frac{{\left[\text{X}/\text{Mn}\right]}_{\text{sample}}}{{\left[\text{X}/\text{Mn}\right]}_{\text{crust}}}, \end{array}$$

where [X/Mn]_sample_ and [X/Mn]_crust_ refer to the ratios of mean concentrations (mg kg^−1^) of the target metal and Mn in the soil and continental crust, respectively [31].

The index of geoaccumulation (*I*_geo_) enables the assessment of contamination by comparing the current and preindustrial concentrations [32]. It can also be applied to the assessment of soil contamination. It is calculated using the following relationship:

(2) $$\begin{array}{c} I_{\text{geo}}=\log_{2}\left(\frac{C_{n}}{1.5B_{n}}\right), \end{array}$$

where *C*_*n*_ is the mean concentration of the element in the examined soil and *B*_*n*_ is the geochemical background value in the crust. The factor 1.5 was introduced to minimize the effect of possible variations in the background values, which may be attributed to lithogenic variations. In the present paper, the modified calculation based on the equation given by Loska et al. [33] was applied, where *C*_*n*_ denoted the concentration of a given element in the examined soil and *B*_*n*_ denoted the concentration of the element in the Earth's crust [34, 35]. Here the focus is on the concentration obtained and the concentration of elements in the Earth's crust because chemical composition of soil is related to the one of the crust. The assessment of soil contamination was also carried out using the contamination factor (*C*_*f*_) and degree of contamination (*C*_deg⁡_). In the version suggested by Hakanson [36], an assessment of soil contamination was carried by using the following relationship:

(3) $$\begin{array}{c} C_{f}^{i}=\frac{C_{i}}{C_{n}^{i}}. \end{array}$$

Here *C*_*i*_ and *C*_*n*_^*i*^, refer to the mean content of metals from at least five sampling sites and the preindustrial soil, respectively. “*n*” represents number of contamination factors involved in the sum. The *C*_*f*_ is the single element index. The sum of contamination factors for all elements examined represents the contamination degree (*C*_deg⁡_) of the environment which is calculated as follows:

(4) $$\begin{array}{c} C_{\deg}=\overset{i=n}{\underset{i=1}{\sum }}C_{f}^{i}. \end{array}$$

In the present study, a modification of the factor as applied by Loska et al. [33] that used the concentration of the elements in the Earth's crust as a reference value, was also used like other indices.

## 3. Results and Discussion

### 3.1. Seasonal Variations

**Figure 2:**
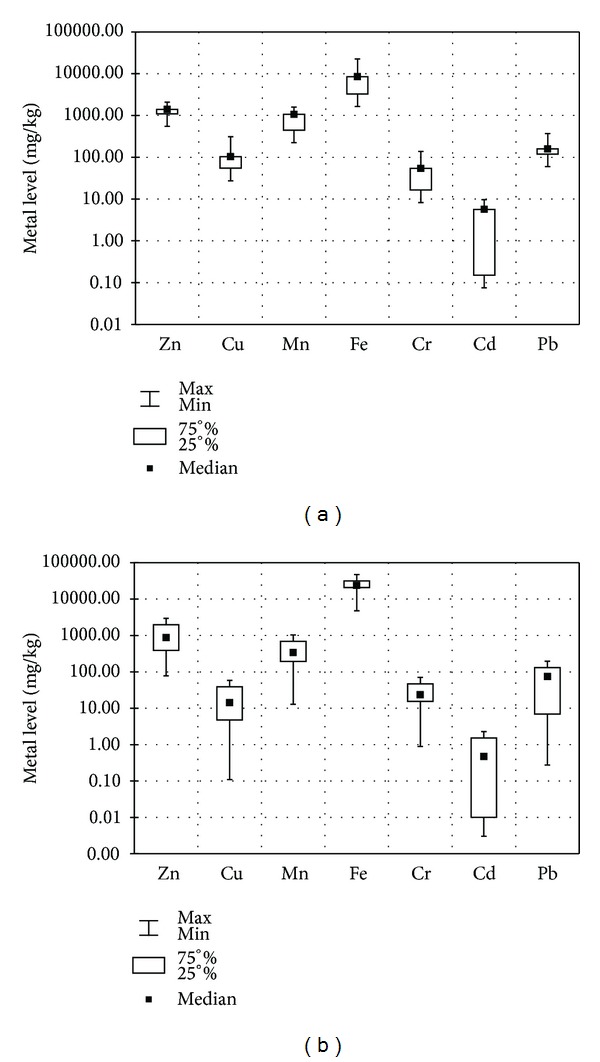
Quartile distribution of selected metals in the soil samples during summer (a) and winter (b).

Results pertaining to the metal distribution in soil during summer and winter seasons are given in (Table 1) and their quartile distribution is shown in (Figure 2). The selected soil samples were found to be strongly acidic in nature showing mean pH values from 4.57 to 6.20 and organic matter ranged from 4.03% to 5.50%. From the data, Fe, Zn, and Mn with mean values of 24291, 937.50, and 338.20 mg kg^−1^, respectively, were the dominant metals in the acid extract of soil samples during summer season, followed by Pb (66.25 mg kg^−1^) and Cr (28.06 mg kg^−1^), exhibiting relatively low concentrations. During summer the concentrations of Cu (16.75 mg kg^−1^) and Cd (0.48 mg kg^−1^) in the soil samples were of the lowest levels [13]. The highest dispersion in terms of standard deviation (SD) and standard error (SE) values during summer was exhibited by Zn, Mn, Fe, and Pb. The quartile distribution of metal concentrations in acid extract is shown in Figure 2(a), where Fe and Cr revealed very narrow distribution while the rest of the metals almost showed random and broad distribution.

The data during winter in Table 1 showed that on the average basis, Fe (10351.83 mg kg^−1^), Zn (1507.79 mg kg^−1^), and Mn (1006.92 mg kg^−1^) were among the dominant metals in the acid extract, followed by Pb (187.18 mg kg^−1^), Cu (123.30 mg kg^−1^), and Cr (69.49 mg kg^−1^). Cd showed the lowest mean concentration of all the metals during winter. In general, most of the metals were significantly higher in winter, which is due to climatic variation during summer and winter. Organic matter content is also the most dominant factor controlling the concentration and retention of these elements in the soils. High organic matter plays an important role in soil structure, water retention, cation exchange, and formation of complexes [37]. The phenomena of high metal concentration in soils during winter were also explained by Niskavaara et al. [38], where they observed that during late autumn and winter the debris from the dying vegetation is accumulated on the soil, increasing the concentrations of all these components. Similar to our findings, Iqbal and Shah [13] reported that most of the precipitation was observed in summer which partially removes the soluble metal contents from the soil, whereas winter mostly remained dry thereby accumulating the deposited metal contents in the soil. The quartile distribution of metal concentration in soils during winter is shown in Figure 2(b), where Zn and Pb cover up narrow distribution with overlapping lower and upper quartiles. During winter, the selected metals Mn, Fe, and Cr showed relatively symmetric distribution, while the rest of the metals showed random and asymmetric distribution. It can be seen in Table 1 that heavy metals were in the following order Fe > Zn > Mn and showed the highest mean concentrations, typically found in pastureland and agricultural soils [39]. The elevated concentration of Mn in the acid extract of the soil during winter is associated with clay contents as in loamy soils and in agricultural soils the concentration was from 20–10,000 mg kg^−1^ [40]. The average levels of Zn, Cu, Cr, Cd, and Pb were considerably higher during winter. The quartile distribution of metal levels during summer and winter manifested comparatively narrow distribution for Fe, Zn, and Pb; however, Cd exhibited broad range and predominantly non-Gaussian distribution.

In the present study, the average metal levels were compared with the values reported elsewhere. The calculated mean levels of Zn in our study were higher in most of the reported levels except in Nigeria [40], whereas the average levels of Cu were lower than the reported world values and comparable to that reported from Guwahati, India [41]. Mean levels of Mn in the present study were found to be lower than those reported from Abakaliki area, Nigeria [40]. The average concentration of Fe in the soils was higher than Shandong, China [42] and Rawal Lake, Pakistan [13] and are significantly lower than Yixing, China [43], Vales, Macedonia [44], Yocsina, Argentina [45], Central Victoria, Australia [46], and Zagreb, Croatia [47]. Nonetheless, the mean concentration of Cr found in the current study was significantly lower than that reported from Gorges area, China [27], Yangzhong, China [5], Pearl River Delta, China [7], Guwahati, India [42], Adana, Turkey [48], Vales, Macedonia [44], and Multan, Pakistan [49]. Mean concentration of Cd was obtained considerably comparable to that reported from Guanting Reservoir, China [50], Yangzhong, China [5], Pearl River Delta, China [7], Rawal Lake (Winter), Pakistan [13], Annaba, Algeria [51], and Vales, Macedonia [44], whereas it was significantly lower than reported from Guwahati, India [42]. The estimated levels of Pb in soils were comparable to those reported from Guanting Reservoir, China [50], Guwahati, India [42], whereas they were significantly lower than reported from Vales, Macedonia [44] and Abakaliki area, Nigeria [40].

### 3.2. Multivariate Analysis for Source Identification of Heavy Metal

Principal Component Analysis (PCA) using varimax-normalized rotation to maximize sum of the variance of factor coefficients showed that 72.04% of the total variance is explained by two VF in summer and 82.21% by three VF in winter (Table 2). In summer, VF1 showed 47.82% of the total variance and has the high negative factor loadings for Zn (−0.75), Cu (−0.76), Fe (−0.72), and Cr (−0.79). These VFs identified metals, which are associated with different anthropogenic activities. VF2 accounted for 24.22% of the total variance and reflected nonsignificant loadings for any metal. In winter, VF1 explains 41.43% of the total variance and revealed significant negative loadings for Zn (−0.74), Cu (−0.88), Fe (−0.87), and Cr (−0.72). Zn, Cu, Fe and Cr sources can be related either to their use in agriculture or in various industrial process. The enrichment of Cu was most likely related to the high application of agrochemicals used to improve production and quality [52]. Cu can also be found in N fertilizers and some kinds of pesticides and germicides. Irrigation with industrial and livestock sewage are usage of sullage and atmospheric deposition are also possible causes for the enrichment in the studied vegetable soils [53]. The concentrations of Cd, Cu, and Zn can be associated with several decades of intensive cropping with high agrochemicals, especially fertilizer inputs. It was found that the land use patterns had significantly different accumulation effects on the heavy metals of Cr, Cu, Cd, and Zn. In general, external sources of heavy metals accessing the soil are mainly from irrigation, solid waste, pesticides, fertilizers, atmospheric deposition, and so forth [54]. Factor 2 accounted for 25.04% of total variance and revealed significant loadings for Pb (−0.724). Despite the sharp increase of unleaded fuel utilization in European countries, the level of Pb in urban soils still is high due to the nondegradability of metal [55, 56]. The previous studies also showed that the Pb concentrations in top 50 cm of forest soils are increasing by ca. 0.2% annually through deposition of atmospheric contamination in Sweden [57]. Moreover, in pesticides the elevated concentration of Pb was recorded by Gimeno-García et al. [58]. However, in the so-called developing countries, leaded gasoline is still widely used. For instance, in Algeria, 89% of the gasoline consumption is leaded (http://www.mem-algeria.org/francais/index.php?page=le-marche-algerien). Therefore, the location of the soils contaminated by Pb, together with the absence of high concentrations in the surroundings of the metallurgical plant (in principal as well as in additional samples), shows that road traffic and atmospheric deposition are the most likely sources of Pb in these soils. 

**Figure 3:**
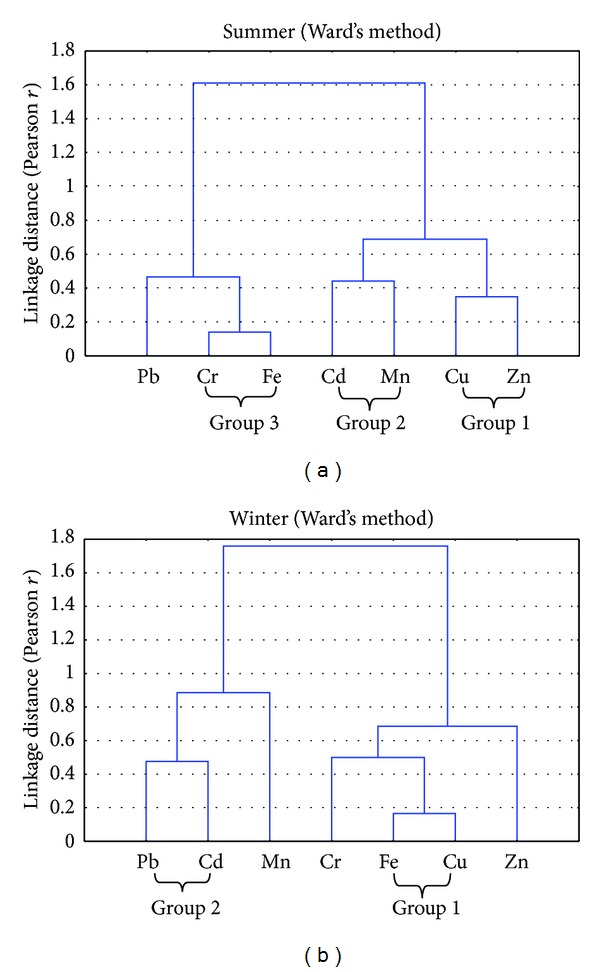
Cluster analyses of selected metals in soil samples during summer (a) and winter (b).

Varimax factor (VF) 3 accounted for 15.75% of the total variance and reflected nonsignificant loadings for any metal. Hierarchical Cluster Analysis (HACA) identified three groups of association between metals in summer and two in winter Figure 3. In summer, group-1 consists of two metals Zn and Cu, suggesting all these metals are from the same source. The presence of trace elements in the slurries has been reported for several years [59]. The applications of swine manure to the agricultural fields are the main source of Cu and Zn enrichment [60]. Fertilizers, superphosphates contain the highest level of Cu and Zn as impurities [58]. Group-2 consists of two metals Mn and Cd, possibly originating from pesticides application. According to [58], the highest concentration of Mn and Cd was found in herbicides and fertilizers. Fe and Mn mainly serve as an indirect marker of the Fe/Mn oxide content in soils, which are known to affect the retention and chemical behavior of heavy metals in soils [61]. High Mn concentration is also associated with clay content as in loamy soils [40]. Group-3 comprises metals (Cr and Fe), which are present in natural soils; these elements are derived from the weathering of parent material and subsequent pedogenesis [5]. While in winter two groups were identified. Group-1 consists of Cu and Fe which may be related to the agriculture activities. Group-2 consists of Pb and Cd and may be due to the industrial activities. Correlation analysis (Table 3) also indicated very similar intermetals relationship (Cu-Zn, Cr-Zn, Mn-Cu, Cd-Cu, Cd-Mn, Cr-Fe, Pb-Fe, and Pb-Cr) in summer for different soil sampling points. VF 1 (Zn, Cu, Fe, Cr) of FA/PCA additionally strengthened these interrelationships for summer. Correlation analysis of winter sampling indicated that metals Fe-Cu, Pb-Cd, Cu-Zn and Cr-Fe have strong correlations and these correlation were also supported by the VF1 (Zn, Cu, Fe, and Cr) and VF2 (Pb).

### 3.3. Risk Assessment

**Figure 4:**
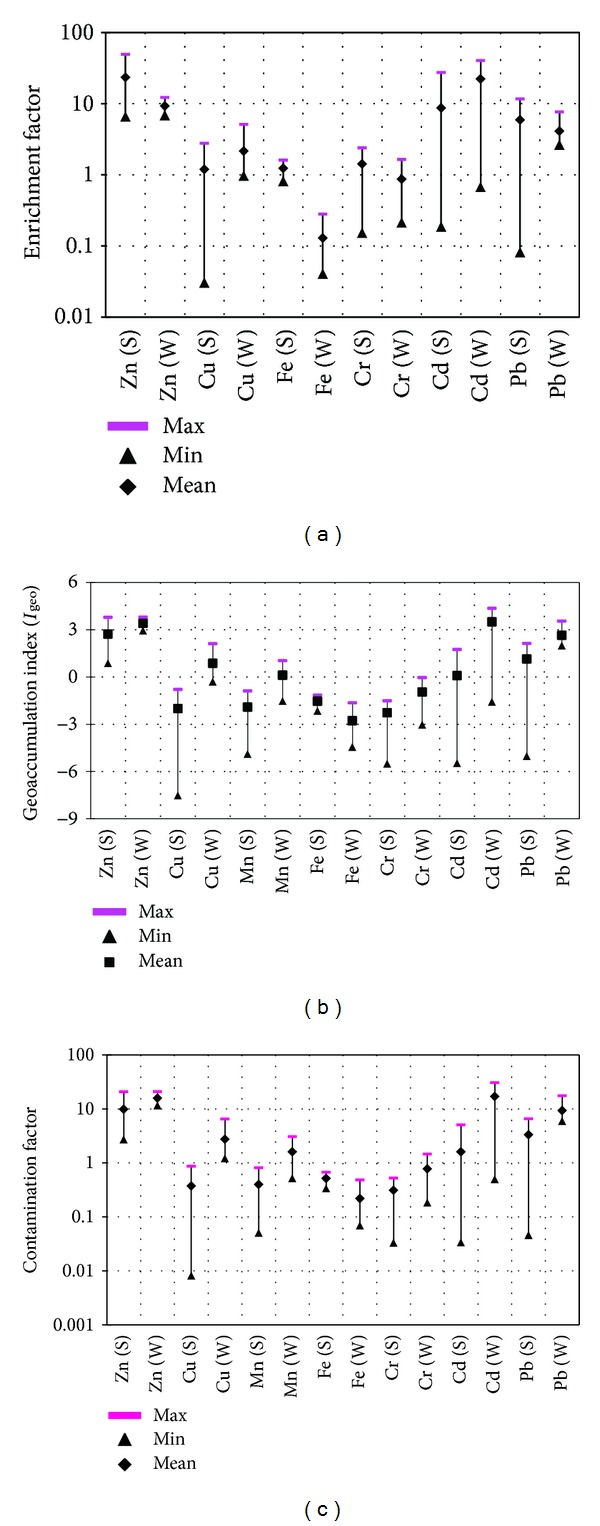
Summary of (a) enrichment factor, (b) geoaccumulation index, and (c) contamination factor for selected metals in the soil samples during summer (S) and winter (W).

To identify anomalous metal contributions and to assess anthropogenic intrusions of the metals in soils, geochemical normalization has been used to calculate enrichment factor (EF) [23]. EF values were interpreted as suggested by Sutherland [62]. The reference element is assumed to have little variability of occurrence and is present in trace concentration in the examined environment. It is also possible to use a geochemically characteristic element which is present in the environment in the large concentration but is characterized by none of these effects, that is, synergism or antagonism towards the examined element. Elements which are most often used as reference ones are Sc, Mn, Al, and Fe [63]. In the present study, Mn is used as the crustal reference element in EF calculations because it is one of the largest components of soil and because of the ease of determination of this element.  Figure 4(a) shows the minimum, mean, and maximum EF values of the selected metals in acid extract of the soils during summer and winter seasons. During summer, the mean EF values of Zn and Pb were above 20 and 5, respectively. The EF values for the rest of the metals were less than 2 and 3. The highest EF values for Zn was 23.42, indicating that this metal was highly enriched in soil. Pb with EF value 5.88 showed significant enrichment in the soil. During winter, the mean EF values of Cd and Zn were greater than 20 and 5, respectively; those of Cu and Pb were between 2 and 5; and those of Fe and Cr were less than 2. The highest EF value for Cd was 22.23, signifying that Cd was highly enriched in the soil, while Zn with EF value 9.28 was significantly enriched in soil. In general, the mean EF values of Zn classified the soil as highly enriched during summer and significantly enriched during winter, whereas those of Cd classified the soil as very highly enriched during winter and moderately enriched during summer.

Geoaccumulation index (*I*_geo_) was also calculated to assess the contamination levels of selected metals in soil (Table 4). The contamination level is assessed by comparing present concentration with preindustrial levels. Concentrations of geochemical background were multiplied each time by a constant factor 1.5 in order to allow content fluctuations of metals in the environment as well as very small anthropogenic influences. The *I*_geo_ minimum, mean, and maximum values of selected metals in acid extract of the soil during summer and winter are presented in Figure 4(b). The mean *I*_geo_ values of Pb and Zn showed moderately to heavily contamination, respectively. The rest of the metals Cu, Mn, Fe, and Cr revealed uncontamination to the soils. The average *I*_geo_ values for Cd indicated that the soil was uncontaminated in the summer and heavily contaminated during winter. The remaining metals showed almost similar behavior in both seasons. During summer, the highest values for Pb and Zn classified the soil as moderately to heavily contaminated, while in the winter, the values for Pb, Zn, and Cd classified the soil as moderately to heavily contaminated, respectively.

The assessment of soil contamination was also done using the contamination factor and degree as suggested by Loska et al. [33] and Hakanson [36]. The (*C*_*f*_) is the single element index; the sum of contamination factors for all elements examined represents the contamination degree (*C*_deg⁡_) of the environment.  Figure 4(c) shows the minimum, mean, and maximum contamination factor (*C*_*f*_) values of the individual metals in acid extract of the soil during summer and winter. In summer, on the basis of average *C*_*f*_ values, the soil was classified as very highly contaminated with Cd and Zn, considerably contaminated with Pb, and as least contaminated with Cu, Mn, Fe, and Cr. The highest *C*_*f*_ values for Cd, Pb, and Zn were 15.01, 9.87, and 3.31 indicating that the soil was very highly contaminated. However, during winter the soil was classified as highest *C*_*f*_ values for Cd and Zn were 16.96 and 15.87, indicating that the soil was very highly polluted. However, the soil was classified as low contaminated with Fe and Cr. In general, the mean *C*_*f*_ values for Cu categorized the soil as moderately contaminated in winter season, whereas those of Pb, Cd, and Zn graded the soil as considerable contaminated to very highly contaminated during both seasons. The mean *C*_deg⁡_ values (Table 5) showed metal concentration in soil during summer is 16.38, indicating considerable contamination, whereas during winter the *C*_deg⁡_ value is 47.10, showing very high degree of contamination. The maximum *C*_deg⁡_ during summer and winter are 35.18 and 79.38, respectively, denoted very high contamination.

## 4. Conclusions

The present study illustrated obvious seasonal variations of the selected heavy metal contents in soil samples. The distribution and covariations of selected metals in soils exhibited seasonal variation. Multivariate analysis revealed significant anthropogenic, point and nonpoint pollution of selected metals in the watershed of the Siling reservoir. The application of the enrichment factor, index of geoaccumulation, and contamination factor enabled us to find elevated contents of some toxic metals, which indicated from moderate to high contamination in the soil samples during summer and winter seasons. Low soil pH and high organic matter contents were found to enhance the leaching of some elements from the soil into water-bearing formations. Consequently, in such conditions toxic metals content may also increase in water sources, especially in Siling reservoir. Moreover, we also found that quality and quantity of applied fertilizers were the important sources leading to different accumulations of heavy metals in soils under the studied land use patterns. Hence, this situation may aggravate the risk to environment in general and specifically to human health in particular. It is, therefore, suggested that the application of agro-based chemical fertilizers and pesticides with high heavy metals content should be avoided to keep high quality soils for sustainable use in the reservoir watershed. Further studies are required to obtain adequate knowledge of the pollution levels and their environmental consequences in the region for better watershed management.

## Figures and Tables

**Table 1:** Mean concentration (mg kg^−1^) of metals in soil during summer and winter seasons in Siling reservoir.

	Summer					Winter				
	Min	Max	Mean	SD	SE	Min	Max	Mean	SD	SE
Zn	258.9	1966	937.50	588.0	151.8	1096	1988	1507.79	281.0	72.54
Cu	0.367	38.98	16.75	12.96	3.347	54.62	292.1	123.30	59.69	15.41
Mn	42.78	691.8	338.20	196.2	50.67	443.7	1507	1006.92	333.2	86.02
Fe	15870	31586	24291	4814	1243	3250	22695	10351.83	5140	1327
Cr	2.967	47.23	28.06	14.99	3.871	16.45	131.4	69.49	43.07	11.12
Cd	0.010	1.517	0.480	0.469	0.121	0.150	9.23	5.09	3.209	0.829
Pb	0.917	131.1	66.25	51.74	13.36	118.8	349.8	187.18	67.88	17.53

**Table 2:** Principal component loading of selected metals in the soil samples.

	Summer		Winter		
	PC 1	PC 2	PC 1	PC 2	PC 3
Eigen value	3.347	1.696	2.900	1.753	1.103
% total variance	47.819	24.223	41.426	25.038	15.75
% cumulative variance	47.819	72.042	41.426	66.464	82.22
Zn	*−0.749*	−0.165	*−0.728*	−0.061	0.441
Cu	*−0.757*	−0.424	*−0.871 *	0.354	0.114
Mn	−0.619	−0.538	0.121	−0.697	−0.514
Fe	*−0.722*	0.512	*−0.863 *	0.373	−0.290
Cr	*−0.785*	0.540	*−0.719 *	−0.142	−0.575
Cd	−0.492	−0.659	−0.348	−0.675	0.465
Pb	−0.671	0.460	−0.462	*−0.724 *	0.043

Italic values are significant at *P* = 0.05.

**Table 3:** Correlation coefficient (*r*) matrix of selected metals in soil during summer (below the diagonal) and winter (above the diagonal).

	Zn	Cu	Mn	Fe	Cr	Cd	Pb
Zn	1	***0.61***	−0.08	0.51	0.18	0.37	0.32
Cu	***0.65***	1	−0.37	***0.83***	0.46	0.11	0.18
Mn	0.44	***0.55***	1	−0.18	0.17	0.13	0.30
Fe	0.30	0.27	0.20	1	***0.71***	−0.08	0.08
Cr	***0.52***	0.36	0.11	***0.86***	1	0.19	0.38
Cd	0.30	***0.55***	***0.56***	0.15	0.10	1	***0.52***
Pb	0.33	0.29	0.30	***0.58***	***0.65***	−0.02	1

Bold italic values are significant at *P* < 0.05.

**Table 4:** Description of enrichment factor (EF), contamination factor (*C*_*f*_), geoaccumulation index (*I*_geo_), and degree of contamination (*C*_deg⁡_) (Hakanson 1980 [36]; Sutherland 2000 [62]).

Value	Soil quality
EF < 2	Deficiency to minimal enrichment
2 < EF < 5	Moderate enrichment
5 < EF < 20	Significant enrichment
20 <EF < 40	Very high enrichment
40 < EF	Extremely high enrichment

*C*_*f*_ < 1	Low contamination factor indicating low contamination
1 ≤ *C*_*f*_ < 3	Moderate contamination factor
3 ≤ *C*_*f*_ < 6	Considerable contamination factor
6 ≤ *C*_*f*_	Very high contamination factor

*I*_geo_ < 0	Practically uncontaminated
0 < *I*_geo_ < 1	Uncontaminated to moderately contaminated
1 < *I*_geo_ < 2	Moderately contaminated
2 < *I*_geo_ < 3	Moderately to heavily contaminated
3 < *I*_geo_ < 4	Heavily contaminated
4 < *I*_geo_ < 5	Heavily to extremely contaminated
5 < *I*_geo_	Extremely contaminated

*C*_deg⁡_ < 8	Low degree of contamination
8 ≤ *C*_deg⁡_ < 16	Moderate degree of contamination
16 ≤ *C*_deg⁡_ < 32	Considerable degree of contamination
32 ≤ *C*_deg⁡_	Very high degree of contamination

**Table 5:** Summary of contamination factor and degree for metals in the soil during summer and winter.

Element	Summer			Winter		
	Min	Max	Mean	Min	Max	Mean
Zn	2.73	20.70	9.87	11.53	20.93	15.87
Cu	0.01	0.87	0.37	1.21	6.49	2.74
Mn	0.05	0.81	0.40	0.52	1.76	1.18
Fe	0.34	0.67	0.51	0.07	0.48	0.22
Cr	0.03	0.52	0.31	0.18	1.46	0.77
Cd	0.03	5.06	1.60	0.50	30.77	16.96
Pb	0.05	6.55	3.31	5.94	17.49	9.36
*C*_deg⁡_	**3.23**	**35.18**	**16.38**	**19.96**	**79.38**	**47.10**
